# Ammonium 4,4-difluoro-1,3,2-dithia­zetin-2-ide 1,1,3,3-tetra­oxide

**DOI:** 10.1107/S1600536812024221

**Published:** 2012-06-02

**Authors:** Maik Finze, Guido J. Reiss

**Affiliations:** a Institut für Anorganische Chemie, Julius-Maximilians-Universität Würzburg, Am Hubland D-97074, Würzburg, Germany; b Institut für Anorganische Chemie und Strukturchemie, Lehrstuhl II: Material- und Strukturforschung, Heinrich-Heine-Universität Düsseldorf, Universitätsstrasse 1, D-40225 Düsseldorf, Germany

## Abstract

The asymmetric unit of the title compound, NH_4_^+^·CF_2_NO_4_S_2_^−^, consists of two crystallographically independent ammonium cations and two 4,4-difluoro-1,3,2-dithia­zetin-2-ide 1,1,3,3-tetra­oxide anions all located in general positions. The S—C—S—N rings of both crystallographically independent anions are almost planar, with the N atom bent out of the plane by 9.82 (5) and 12.82 (4)°. The structure was determined from a crystal twinned by inversion, with refined components in the ratio 0.73 (4):0.27 (4). Anions and cations are connected *via* hydrogen bonds (N—H⋯O and N—H⋯N) to form a three-dimensional framework. This framework is composed of two different layers parallel to the *ab* plane, which are built by the ammonium cations on the one hand and the complex cyclic anions on the other.

## Related literature

For general aspects of the chemistry of fluorinated sulfonimides and their salts, see: Antoniotti *et al.* (2010 ▶); Foropoulos & DesMarteau (1984 ▶); Popov *et al.* (2011 ▶); Vij *et al.* (1997 ▶); DesMarteau (1995 ▶). For the synthesis and chemistry of the title compound, see: Jüschke *et al.* (1997 ▶). For related structures, see: DesMarteau *et al.* (1992 ▶); Davidson *et al.* (2003 ▶). For similar layered ammonium salts, see: Reiss (2002 ▶); Plizko & Meyer (1998 ▶); Bucholz & Mattes (1988 ▶).
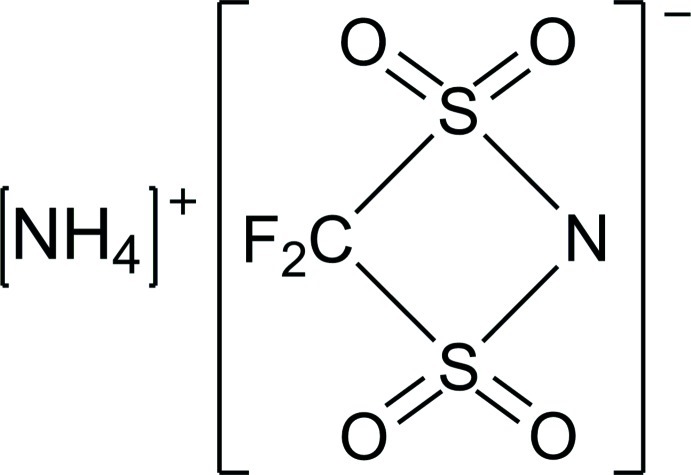


## Experimental

### 

#### Crystal data

NH_4_^+^·CF_2_NO_4_S_2_^−^
*M* *_r_* = 210.18
Orthorhombic, 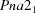

*a* = 11.28642 (13) Å
*b* = 10.98496 (14) Å
*c* = 10.58826 (12) Å
*V* = 1312.74 (3) Å^3^
*Z* = 8
Mo *K*α radiation
μ = 0.82 mm^−1^
*T* = 100 K
0.30 × 0.25 × 0.20 mm

#### Data collection

Oxford Diffraction Xcalibur Eos diffractometer
Absorption correction: multi-scan (*CrysAlis PRO*; Oxford Diffraction, 2009 ▶) *T*_min_ = 0.922, *T*_max_ = 1.000
25817 measured reflections
3823 independent reflections
3778 reflections with *I* > 2σ(*I*)
*R*_int_ = 0.028

#### Refinement

*R*[*F* ^2^ > 2σ(*F* ^2^)] = 0.019
*wR*(*F* ^2^) = 0.049
*S* = 1.00
3823 reflections
227 parameters
9 restraints
All H-atom parameters refined
Δρ_max_ = 0.42 e Å^−3^
Δρ_min_ = −0.23 e Å^−3^
Absolute structure: Flack (1983 ▶), 1816 Friedel pairs
Flack parameter: 0.27 (4)

### 

Data collection: *CrysAlis PRO* (Oxford Diffraction, 2009 ▶); cell refinement: *CrysAlis PRO*; data reduction: *CrysAlis PRO*; program(s) used to solve structure: *SHELXS97* (Sheldrick, 2008 ▶); program(s) used to refine structure: *SHELXL97* (Sheldrick, 2008 ▶); molecular graphics: *DIAMOND* (Brandenburg, 2011 ▶); software used to prepare material for publication: *publCIF* (Westrip, 2010 ▶).

## Supplementary Material

Crystal structure: contains datablock(s) I, global. DOI: 10.1107/S1600536812024221/pk2414sup1.cif

Structure factors: contains datablock(s) I. DOI: 10.1107/S1600536812024221/pk2414Isup2.hkl

Additional supplementary materials:  crystallographic information; 3D view; checkCIF report

## Figures and Tables

**Table 1:** Hydrogen-bond geometry (Å, °)

*D*—H⋯*A*	*D*—H	H⋯*A*	*D*⋯*A*	*D*—H⋯*A*
N1*A*—H1⋯O1	0.86 (1)	2.11 (2)	2.9044 (17)	153 (2)
N1*A*—H3⋯N1^i^	0.87 (1)	2.20 (1)	3.0395 (19)	165 (2)
N1*A*—H4⋯O8^ii^	0.85 (1)	2.13 (1)	2.9657 (17)	166 (2)
N2*A*—H5⋯O5	0.87 (1)	2.04 (1)	2.8985 (16)	175 (3)
N2*A*—H6⋯N2^iii^	0.86 (1)	2.20 (2)	3.0068 (18)	157 (3)
N2*A*—H7⋯O2^iv^	0.86 (1)	2.03 (2)	2.8467 (17)	158 (3)
N2*A*—H8⋯O7^v^	0.86 (1)	2.13 (2)	2.8832 (17)	146 (2)

Symmetry codes: (i) 
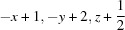
; (ii) 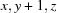; (iii) 
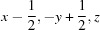
; (iv) 
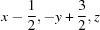
; (v) 
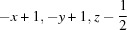
.

**Fig. 1.**
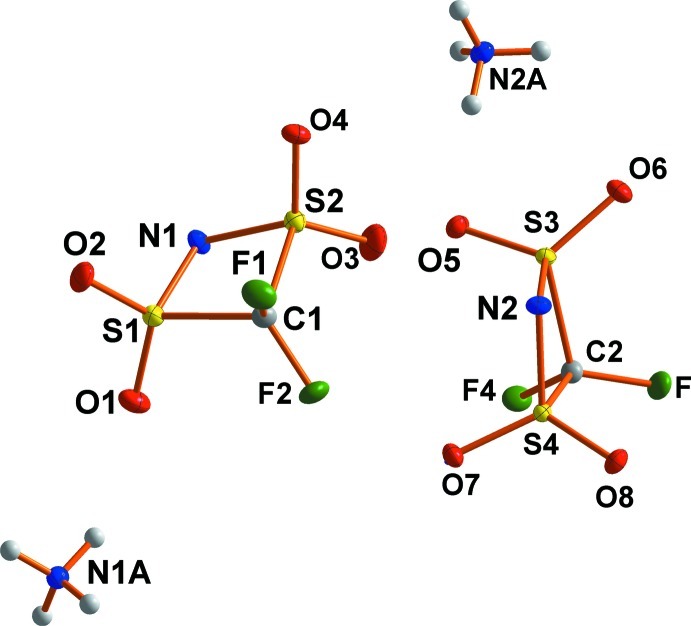
The asymmetric unit of the title compound, with displacement ellipsoids drawn at the 50% probability level; H atoms are drawn as spheres of arbitrary radius.

**Fig. 2.**
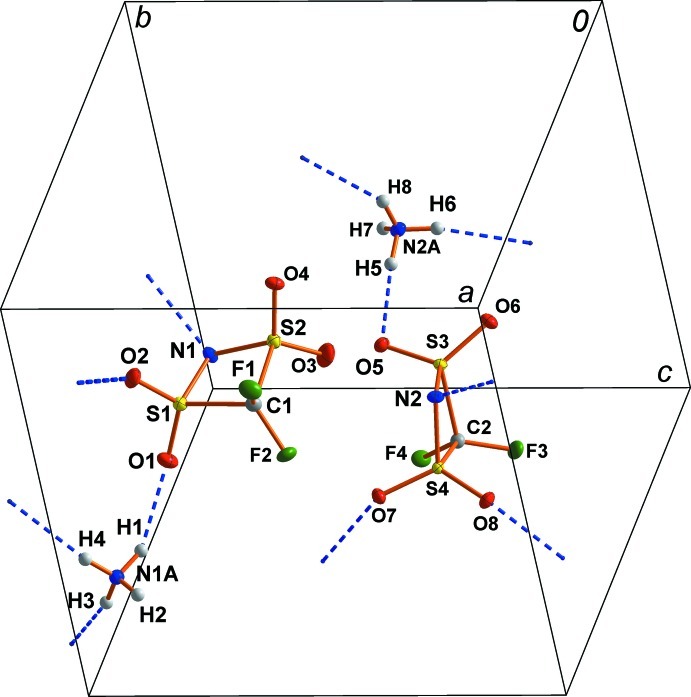
The asymmetric unit of the title compound positioned in the unit cell; H-atoms are drawn with arbitrary radii; hydrogen bonds shown as broken lines.

**Fig. 3.**
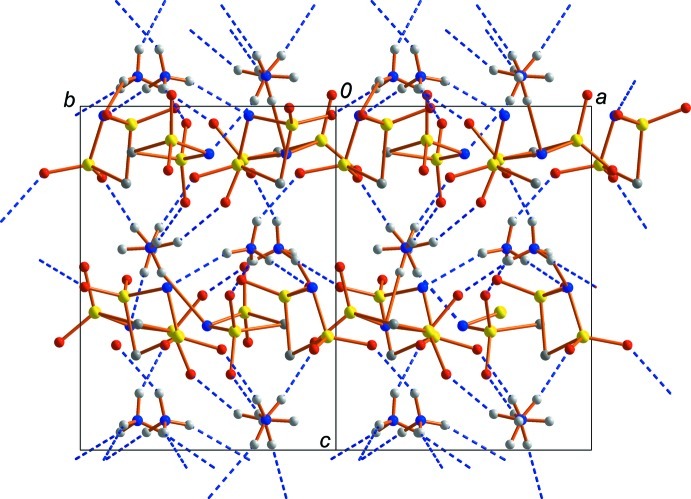
Layered structure of the title compound with view along [110] (ball and stick type model with arbitrary atom radii; fluorine atoms are omitted for clarity; hydrogen bonds shown as broken lines).

## supplementary crystallographic information

### Comment

Cyclic sulfonimides of the general formula *cyclo*-[(F_2_C)_n_(SO_2_)_2_NH] and the corresponding anions are relevant because of a number of potential applications in various fields, *e.g.* electrochemistry and organic chemistry similar to the non-cyclic sulfonimides (*R*^F^SO_2_)_2_NH (Popov *et al.*, 2011; Antoniotti *et al.*, 2010; Vij *et al.*, 1997; Jüschke *et al.*, 1997; DesMarteau, 1995; DesMarteau *et al.*, 1992; Foropoulos & DesMarteau, 1984). The synthesis of the ammonium salt of the smallest member of the cyclic sulfonimides was first reported almost 20 years ago starting from the disulfonylfluoride FO_2_SCF_2_SO_2_F and ammonia (Jüschke *et al.*, 1997). The conversion of the ammonium salt to result in the potassium and rubidium salt with KOH and Rb_2_CO_3_, respectively and the crystal structures of these alkali metal salts were described, as well.

The title compound ammonium 4,4-difluoro-1,3,2-dithiazetin-2-ide 1,1,3,3-tetraoxide (Fig. 1) crystallizes in the orthorhombic non-centrosymmetric space group *Pna*2_1_ with two independent cations and anions. The ammonium containing title structure is isotypical to the corresponding potassium salt whereas the rubidium salt crystallizes in the monoclinic space group *P*2_1_/*c* (Jüschke *et al.*, 1997). The S–C–S–N ring is almost planar and the nitrogen atom is bent out of the plane by 9.82 (5) and 12.82 (4)°, respectively in the two anions. The bond lengths and angles of the cyclic anion in its [NH_4_]^+^ and K^+^ salt are very similar, whereas slightly larger deviations are found for the Rb^+^ salt. Medium to weak hydrogen bonds connect the [NH_4_]^+^ cations and the cyclic anions to form a three-dimensional framework (Fig. 2). The cyclic anion accepts hydrogen bonds by the oxygen atoms of the SO_2_ group and by its amide nitrogen atom. The N—H···N hydrogen bonds are, according to the derived distances weaker than the N—H···O bonds. In general N—H···N hydrogen bonds are rare in structurally related salts. For example in the structure of the ammonium triflamide salt, [NH_4_][F_3_C—SO_2_—N—SO_2_—CF_3_] no hydrogen bond between the amide nitrogen and the ammonium counter cation are present (Davidson *et al.*, 2003). The title structure can be understood as a layered material. The cations and anions are found in layers perpendicular to the *c* axis (Fig. 3). The ammonium cations appear ordered as a consequence of their hydrogen bonds. The resulting non-centrosymmetric, layered ammonium salt fits well in the general structural chemistry of ammonium salts with layered structures (*e.g.* Reiss, 2002; Plizko & Meyer, 1998; Bucholz & Mattes, 1988).

### Experimental

The title compound was synthesized according to a literature procedure (Jüschke *et al.*, 1997).

### Refinement

In the final stages of refinement the Flack-parameter indicated inversion twinning. The refinement of the twin components (Flack, 1983; Sheldrick, 2008) gave a ratio of 0.73 (4): 0.27 (4). All hydrogen atom positions were identified in difference syntheses. In the final stages of refinement the hydrogen atom positions of these were refined with their N—H distances softly restrained to one common refined value (0.86 Å) with one common U_iso_ value for each group.

### Crystal data

**Table:**

NH_4_^+^·CF_2_NO_4_S_2_^−^	*F*(000) = 848
*M**_r_* = 210.18	*D*_x_ = 2.127 Mg m^−^^3^
Orthorhombic, *P**n**a*2_1_	Mo *K*α radiation, λ = 0.71073 Å
Hall symbol: P 2c -2n	Cell parameters from 21999 reflections
*a* = 11.28642 (13) Å	θ = 3.2–33.6°
*b* = 10.98496 (14) Å	µ = 0.82 mm^−^^1^
*c* = 10.58826 (12) Å	*T* = 100 K
*V* = 1312.74 (3) Å^3^	Block, colourless
*Z* = 8	0.30 × 0.25 × 0.20 mm

### Data collection

**Table:**

Oxford Diffraction Xcalibur Eos diffractometer	3823 independent reflections
Radiation source: fine-focus sealed tube	3778 reflections with *I* > 2σ(*I*)
Graphite monochromator	*R*_int_ = 0.028
Detector resolution: 16.2711 pixels mm^-1^	θ_max_ = 30.0°, θ_min_ = 3.2°
ω scans	*h* = −15→15
Absorption correction: multi-scan (*CrysAlis PRO*; Oxford Diffraction, 2009)	*k* = −15→15
*T*_min_ = 0.922, *T*_max_ = 1.000	*l* = −14→14
25817 measured reflections	

### Refinement

**Table:**

Refinement on *F*^2^	Secondary atom site location: difference Fourier map
Least-squares matrix: full	Hydrogen site location: difference Fourier map
*R*[*F*^2^ > 2σ(*F*^2^)] = 0.019	All H-atom parameters refined
*wR*(*F*^2^) = 0.049	*w* = 1/[σ^2^(*F*_o_^2^) + (0.027*P*)^2^ + 0.6*P*] where *P* = (*F*_o_^2^ + 2*F*_c_^2^)/3
*S* = 1.00	(Δ/σ)_max_ = 0.001
3823 reflections	Δρ_max_ = 0.42 e Å^−^^3^
227 parameters	Δρ_min_ = −0.23 e Å^−^^3^
9 restraints	Absolute structure: Flack (1983), 1816 Friedel pairs
Primary atom site location: structure-invariant direct methods	Flack parameter: 0.27 (4)

### Special details

**Table:**

Experimental. Absorption correction: CrysAlisPro, Oxford Diffraction Ltd., Empirical absorption correction using spherical harmonics, implemented in SCALE3 ABSPACK scaling algorithm.
Geometry. All e.s.d.'s (except the e.s.d. in the dihedral angle between two l.s. planes) are estimated using the full covariance matrix. The cell e.s.d.'s are taken into account individually in the estimation of e.s.d.'s in distances, angles and torsion angles; correlations between e.s.d.'s in cell parameters are only used when they are defined by crystal symmetry. An approximate (isotropic) treatment of cell e.s.d.'s is used for estimating e.s.d.'s involving l.s. planes.
Refinement. Refinement of *F*^2^ against ALL reflections. The weighted *R*-factor *wR* and goodness of fit *S* are based on *F*^2^, conventional *R*-factors *R* are based on *F*, with *F* set to zero for negative *F*^2^. The threshold expression of *F*^2^ > σ(*F*^2^) is used only for calculating *R*-factors(gt) *etc*. and is not relevant to the choice of reflections for refinement. *R*-factors based on *F*^2^ are statistically about twice as large as those based on *F*, and *R*- factors based on ALL data will be even larger.

### Fractional atomic coordinates and isotropic or equivalent isotropic displacement parameters (Å^2^)

**Table:**

	*x*	*y*	*z*	*U*_iso_*/*U*_eq_	
S1	0.48379 (3)	0.87892 (3)	0.65469 (3)	0.00949 (7)	
S2	0.35058 (3)	0.70690 (3)	0.59946 (3)	0.00899 (7)	
O1	0.51045 (10)	0.92154 (11)	0.77958 (11)	0.0153 (2)	
O2	0.53141 (10)	0.94654 (10)	0.55027 (12)	0.0180 (2)	
O3	0.28692 (10)	0.63106 (11)	0.68639 (12)	0.0188 (2)	
O4	0.32923 (10)	0.68643 (11)	0.46733 (11)	0.0141 (2)	
C1	0.51223 (12)	0.71376 (13)	0.63358 (13)	0.0101 (3)	
F1	0.58461 (8)	0.68458 (9)	0.53906 (9)	0.01534 (18)	
F2	0.54517 (8)	0.65524 (9)	0.73788 (9)	0.01670 (18)	
N1	0.34538 (11)	0.84878 (11)	0.63851 (13)	0.0126 (2)	
S3	0.48620 (3)	0.31465 (3)	0.55319 (3)	0.00837 (6)	
S4	0.67564 (3)	0.29184 (3)	0.67001 (3)	0.00808 (6)	
O6	0.40654 (9)	0.22416 (10)	0.50504 (11)	0.0135 (2)	
O5	0.45653 (9)	0.43993 (9)	0.52553 (10)	0.0129 (2)	
O7	0.73598 (9)	0.40480 (10)	0.69468 (10)	0.0126 (2)	
O8	0.73416 (9)	0.18093 (10)	0.70717 (11)	0.0128 (2)	
F3	0.47997 (8)	0.20036 (8)	0.77901 (9)	0.01355 (17)	
F4	0.49019 (8)	0.39790 (9)	0.79297 (9)	0.01288 (17)	
N2	0.62391 (11)	0.28395 (11)	0.52747 (12)	0.0104 (2)	
C2	0.51915 (12)	0.30127 (13)	0.72413 (14)	0.0095 (2)	
N1A	0.72362 (11)	0.99695 (12)	0.91184 (12)	0.0122 (2)	
H1	0.6585 (14)	0.9597 (19)	0.895 (3)	0.030 (3)*	
H2	0.7758 (17)	0.9398 (16)	0.916 (2)	0.030 (3)*	
H3	0.715 (2)	1.034 (2)	0.9834 (15)	0.030 (3)*	
H4	0.739 (2)	1.0475 (18)	0.8532 (18)	0.030 (3)*	
N2A	0.22203 (11)	0.44588 (12)	0.41410 (12)	0.0132 (2)	
H5	0.2919 (14)	0.449 (2)	0.448 (2)	0.041 (4)*	
H6	0.201 (2)	0.3720 (13)	0.426 (3)	0.041 (4)*	
H7	0.173 (2)	0.496 (2)	0.449 (2)	0.041 (4)*	
H8	0.230 (2)	0.461 (2)	0.3348 (12)	0.041 (4)*	

### Atomic displacement parameters (Å^2^)

**Table:**

	*U*^11^	*U*^22^	*U*^33^	*U*^12^	*U*^13^	*U*^23^
S1	0.00951 (13)	0.00917 (14)	0.00979 (15)	−0.00032 (10)	−0.00095 (11)	−0.00005 (12)
S2	0.00864 (13)	0.00925 (14)	0.00907 (14)	−0.00050 (11)	0.00066 (11)	0.00012 (12)
O1	0.0149 (5)	0.0167 (5)	0.0143 (5)	0.0003 (4)	−0.0040 (4)	−0.0052 (4)
O2	0.0186 (5)	0.0171 (5)	0.0183 (5)	−0.0020 (4)	0.0028 (4)	0.0068 (5)
O3	0.0179 (5)	0.0187 (5)	0.0199 (6)	−0.0036 (4)	0.0056 (4)	0.0072 (4)
O4	0.0128 (5)	0.0177 (5)	0.0117 (5)	0.0013 (4)	−0.0028 (4)	−0.0041 (4)
C1	0.0096 (6)	0.0106 (6)	0.0101 (6)	0.0015 (4)	−0.0002 (4)	−0.0011 (5)
F1	0.0108 (4)	0.0203 (4)	0.0149 (4)	0.0023 (3)	0.0030 (3)	−0.0056 (3)
F2	0.0204 (4)	0.0158 (4)	0.0139 (4)	0.0033 (4)	−0.0070 (4)	0.0036 (3)
N1	0.0098 (5)	0.0098 (5)	0.0183 (6)	0.0008 (4)	−0.0019 (4)	−0.0037 (5)
S3	0.00810 (13)	0.00965 (14)	0.00735 (14)	0.00008 (11)	−0.00006 (11)	−0.00046 (11)
S4	0.00836 (13)	0.00801 (14)	0.00787 (14)	0.00020 (10)	−0.00012 (11)	0.00033 (11)
O6	0.0124 (5)	0.0157 (5)	0.0123 (5)	−0.0035 (4)	−0.0003 (4)	−0.0038 (4)
O5	0.0141 (5)	0.0111 (5)	0.0133 (5)	0.0026 (4)	−0.0019 (4)	0.0013 (4)
O7	0.0142 (4)	0.0115 (5)	0.0122 (5)	−0.0040 (4)	0.0006 (4)	−0.0013 (4)
O8	0.0129 (4)	0.0121 (5)	0.0133 (5)	0.0035 (4)	0.0008 (4)	0.0036 (4)
F3	0.0140 (4)	0.0138 (4)	0.0128 (4)	−0.0027 (3)	0.0018 (3)	0.0048 (3)
F4	0.0151 (4)	0.0135 (4)	0.0100 (4)	0.0037 (3)	0.0004 (3)	−0.0041 (3)
N2	0.0083 (5)	0.0149 (6)	0.0079 (5)	0.0011 (4)	0.0001 (4)	−0.0009 (4)
C2	0.0103 (6)	0.0091 (6)	0.0091 (6)	−0.0001 (4)	0.0004 (5)	0.0002 (5)
N1A	0.0136 (6)	0.0116 (5)	0.0114 (6)	−0.0006 (4)	0.0000 (4)	0.0007 (4)
N2A	0.0113 (5)	0.0150 (6)	0.0133 (6)	−0.0003 (4)	−0.0009 (5)	0.0037 (5)

### Geometric parameters (Å, º)

**Table:**

S1—O1	1.4347 (12)	S4—O7	1.4394 (11)
S1—O2	1.4363 (12)	S4—O8	1.4406 (11)
S1—N1	1.6060 (12)	S4—N2	1.6206 (12)
S1—C1	1.8559 (14)	S4—C2	1.8596 (15)
S2—O3	1.4344 (12)	F3—C2	1.3274 (16)
S2—O4	1.4373 (12)	F4—C2	1.3285 (16)
S2—N1	1.6135 (13)	N1A—H1	0.860 (11)
S2—C1	1.8614 (14)	N1A—H2	0.862 (11)
C1—F2	1.3308 (16)	N1A—H3	0.865 (11)
C1—F1	1.3311 (16)	N1A—H4	0.852 (12)
S3—O6	1.4340 (11)	N2A—H5	0.866 (12)
S3—O5	1.4463 (11)	N2A—H6	0.855 (12)
S3—N2	1.6135 (12)	N2A—H7	0.862 (12)
S3—C2	1.8535 (15)	N2A—H8	0.860 (12)
			
O1—S1—O2	117.53 (7)	O7—S4—O8	117.55 (7)
O1—S1—N1	111.69 (7)	O7—S4—N2	112.68 (7)
O2—S1—N1	112.86 (7)	O8—S4—N2	111.98 (7)
O1—S1—C1	113.18 (7)	O7—S4—C2	110.21 (6)
O2—S1—C1	110.37 (7)	O8—S4—C2	113.48 (6)
N1—S1—C1	87.36 (6)	N2—S4—C2	87.01 (6)
O3—S2—O4	116.73 (7)	S3—N2—S4	100.29 (7)
O3—S2—N1	112.23 (7)	F3—C2—F4	110.19 (12)
O4—S2—N1	113.23 (7)	F3—C2—S3	115.27 (10)
O3—S2—C1	112.95 (7)	F4—C2—S3	115.03 (10)
O4—S2—C1	111.07 (6)	F3—C2—S4	113.88 (10)
N1—S2—C1	86.95 (6)	F4—C2—S4	116.57 (10)
F2—C1—F1	109.64 (11)	S3—C2—S4	83.92 (6)
F2—C1—S1	114.87 (10)	H1—N1A—H2	104 (2)
F1—C1—S1	115.60 (10)	H1—N1A—H3	108 (2)
F2—C1—S2	114.54 (10)	H2—N1A—H3	112 (2)
F1—C1—S2	116.48 (10)	H1—N1A—H4	110 (2)
S1—C1—S2	83.88 (6)	H2—N1A—H4	112 (2)
S1—N1—S2	101.01 (7)	H3—N1A—H4	111 (2)
O6—S3—O5	116.26 (7)	H5—N2A—H6	103 (3)
O6—S3—N2	113.52 (7)	H5—N2A—H7	113 (3)
O5—S3—N2	112.81 (6)	H6—N2A—H7	112 (3)
O6—S3—C2	114.71 (7)	H5—N2A—H8	108 (2)
O5—S3—C2	108.64 (6)	H6—N2A—H8	111 (3)
N2—S3—C2	87.42 (7)	H7—N2A—H8	111 (2)
			
N1—S1—C1—S2	6.25 (6)	C2—S3—N2—S4	−9.52 (7)
N1—S2—C1—S1	−6.22 (6)	C2—S4—N2—S3	9.49 (7)
C1—S1—N1—S2	−7.31 (7)	N2—S3—C2—S4	8.20 (6)
C1—S2—N1—S1	7.29 (7)	N2—S4—C2—S3	−8.17 (6)

### Hydrogen-bond geometry (Å, º)

**Table:**

*D*—H···*A*	*D*—H	H···*A*	*D*···*A*	*D*—H···*A*
N1*A*—H1···O1	0.86 (1)	2.11 (2)	2.9044 (17)	153 (2)
N1*A*—H3···N1^i^	0.87 (1)	2.20 (1)	3.0395 (19)	165 (2)
N1*A*—H4···O8^ii^	0.85 (1)	2.13 (1)	2.9657 (17)	166 (2)
N2*A*—H5···O5	0.87 (1)	2.04 (1)	2.8985 (16)	175 (3)
N2*A*—H6···N2^iii^	0.86 (1)	2.20 (2)	3.0068 (18)	157 (3)
N2*A*—H7···O2^iv^	0.86 (1)	2.03 (2)	2.8467 (17)	158 (3)
N2*A*—H8···O7^v^	0.86 (1)	2.13 (2)	2.8832 (17)	146 (2)

Symmetry codes: (i) −*x*+1, −*y*+2, *z*+1/2; (ii) *x*, *y*+1, *z*; (iii) *x*−1/2, −*y*+1/2, *z*; (iv) *x*−1/2, −*y*+3/2, *z*; (v) −*x*+1, −*y*+1, *z*−1/2.
